# Case Report: Infantile Bullous Pemphigoid: Triggering by COVID-19 Is Speculative

**DOI:** 10.3389/fmed.2021.760823

**Published:** 2021-11-22

**Authors:** Anna Rosińska-Więckowicz, Magdalena Jałowska, Monika Bowszyc-Dmochowska, Marian Dmochowski

**Affiliations:** ^1^Autoimmune Blistering Dermatoses Section, Department of Dermatology, Poznan University of Medical Sciences, Poznań, Poland; ^2^Cutaneous Histopathology and Immunopathology Section, Department of Dermatology, Poznan University of Medical Sciences, Poznań, Poland

**Keywords:** bullous pemphigoid, infants, autoimmune blistering, COVID-19, RT-PCR

## Abstract

Bullous pemphigoid (BP) is a cutaneous disease triggered by numerous stimuli, where genetic milieu-influenced autoimmunity to hemidesmosomal proteins, namely, BP180 and/or BP230 initiate an inflammation leading to dermal-epidermal junction (DEJ) enzymatic pathological remodelling. Here, to the best of our knowledge, we present the first case of an infantile BP apparently triggered by COVID-19. BP should be included in differential diagnosis of infantile rashes showing blisters or vesicles or both as well as their prodromal and evolutionary lesions. Possible triggers, such as coronavirus disease 2019 (COVID-19), of BP in infancy should be identified and properly dealt with.

## Introduction

Bullous pemphigoid (BP) is an autoimmune blistering dermatosis mediated by the antibodies to skin hemidesmosomal proteins, BP180 and/or BP230. BP has a genetic, autoimmune, and inflammatory background, leading to dermal-epidermal junction (DEJ) remodelling (1, 2).

Generally, BP is a disorder of the elderly, but it can affect children as well (3). Senescence, particularly combined with neurodegeneration, is regarded as a BP inducing process, which probably acts by altering the physiological immune response regulatory mechanisms (4). Still, numerous apparent triggers of BP, plausibly acting as co-triggers, were described in the literature, such as medications, malignancies, vaccinations, and infections (3, 5, 6).

The origins of the coronavirus disease 2019 (COVID-19) pandemic are debatable (7). There are concerns over the impact of the COVID-19 pandemic on the autoimmune blistering diseases (8).

Here, to the best of our knowledge, we present the first case of an infantile BP apparently triggered by COVID-19.

## Case Report

**Figure 1:**
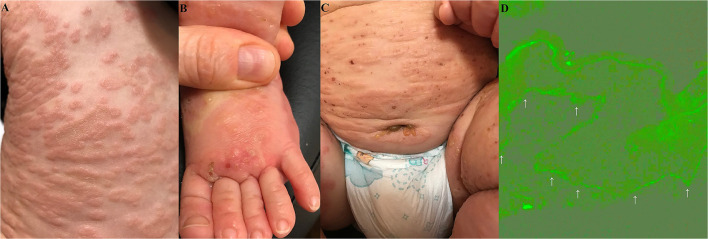
Widespread erythematous plaques on the trunk **(A)**. Vesicles and their evolutionary lesions on an erythematous base on a dorsal surface of a foot **(B)**. The blisters and vesicles around the umbilicus and in the left groin **(C)**. Deposits (indicated by arrows) of IgG1 (+) with an indeterminate pattern along the dermal-epidermal junction (epidermis is at the top of the picture) in direct immunofluorescence of perilesional skin in the periumbilical area are visualised with blue light-emitting diode technology-operated microscopy (original objective magnification × 40) **(D)**.

An 8-month-old infant was referred by a paediatrician to a dermatology outpatient clinic due to disseminated blisters and erosions failing to respond to treatment. The skin lesions were presented for about 5 weeks. According to the mother, the parents and elder brother of the boy had suffered from gastroenterocolitis at that time. All the family members presented fever of 38–38.5°C accompanied by diarrhoea and abdominal pain. On the third day of gastroenterocolitis, our patient started to present non-itchy erythematous plaques on the palms and soles, with solitary vesicles, accompanied by loss of appetite. The patient was consulted by a paediatrician, who suspected the Boston disease with secondary bacterial infection and administered an analgetic (acetaminophen), an antibiotic (amoxycillin with clavulanic acid), topical glucocorticosteroids (fluticasone and hydrocortisone), and dimetindene (oral solution). After 7 days of application, there was no response to that treatment regime. The boy was referred to a hospital, where he was consulted by another paediatrician, who administered clarithromycin. The treatment with clarithromycin was discontinued after few days, as according to the mother, erythematous circular plaques started to spread from palms and soles proximally, affecting forearms, arms, shins, thighs, and the trunk (Figure 1A). Moreover, tense blisters and vesicles on the erythematous background, initially located on distal parts of upper and lower limbs (Figure 1B), were gradually spreading to the periumbilical area (Figure 1C), the whole trunk, and the face. The skin of the scalp and diaper area was spared.

There was no family history of atopic diseases. The boy was breastfed since birth and mother did not have to use any kind of elimination diet, and the diet was expanded according to the guidelines without any disturbing symptoms. A family history of autoimmune blistering skin diseases and chronic diseases was negative. There was no recent history of vaccination prior to the onset of lesions. The boy was vaccinated only on the first day of life (against hepatitis B and tuberculosis), while other mandatory vaccinations were postponed due to mild but frequent oropharyngeal infections, transmitted mostly by older brother of the patient.

Although our patient presented new blisters every day, with some of them turning into oozing erosions covered with yellowish crusts, the boy was in a good general condition, without any additional systemic symptoms, such as fever, weakness, or pruritus. A chest X-ray ordered by a paediatrician showed slight inflammatory lesions pericardially and in the upper lung fields. Abdomen ultrasound did not reveal any abnormalities.

Interestingly, mother of the boy decided to check if the family members had COVID-19 infection, and about 4 weeks after the onset of gastrointestinal symptoms, the pertinent blood tests were performed. It turned out that our patient, older brother, and father all had IgG antibodies against the severe acute respiratory syndrome coronavirus 2 (SARS-CoV-2) (determined by CMIA—chemiluminescent microparticle immunoassay-−235 BAU/ml), while mother of the boy had a positive SARS-CoV-2 reverse transcription PCR (RT-PCR) test.

Our patient was referred to a dermatologist who suspected erythema multiforme or Kaposi varicelliform eruption with secondary infection and prescribed topical treatment with fucidic acid combined with hydrocortisone, cleansing with sodium hypochlorite, and systemic treatment with acyclovir.

As there was no improvement after 2 days of this treatment and the boy started to refuse to eat or drink, the dermatologist decided to refer the infant to a hospital. Laboratory findings showed slightly increased levels of platelets (480 × 10^9^ per L), slight microcytic anaemia, higher levels of eosinophils (7%) in a complete blood count with differential leukocyte count, slightly elevated C-reactive protein (12 mg/dl) with negative procalcitonin (0.04 ng/ml), slightly higher D-dimer (11.64 mg/L), and slightly lower level of serum IgA (24 mg/dl). Culture from blister fluid showed growth of *Streptococcus pyogenes*. The RT-PCR test for the SARS-CoV-2 was negative.

At last, the consulting dermatologist suspected an autoimmune blistering skin disease and took a biopsy from peribullous skin for direct immunofluorescence (DIF). DIF revealed linear deposits of IgG (+), IgG1 (+) (Figure 1D), and C3 (++) along the DEJ. No deposits of IgA, IgM, and IgG4 were found. Multiplex ELISA (Euroimmun, Germany) detected the markedly increased level of IgG antibodies against BP180 (9.81), whereas the levels of antibodies against BP230, desmoglein 1, desmoglein 3, type VII collagen, and envoplakin were within normal range (negative level ratio <1). Thus, BP was diagnosed.

Treatment with systemic methylprednisolone intravenously at a single 40 mg dose, intravenous immunoglobulin (1.5 g/kg bwt, the dose was administered within 2 days), amoxicillin (due to secondary bacterial infection of blisters with *Streptococcus pyogenes*), and a pain reliever (paracetamol) were introduced. A very fast improvement of skin condition was observed during the treatment. After a 5-day hospital stay, our patient was discharged home, and systemic treatment with oral prednisolone and amoxicillin was continued. The doses of prednisone were tapered gradually, about 2.5 mg every 2 weeks, and the treatment was discontinued after 6 weeks. Aggravation of the disease when tapering the dose of prednisone was not observed. With total resolution of lesions that left only discreet post-inflammatory discolorations, the boy remains in good condition.

## Discussion

Of note, periumbilical blisters should be regarded as a clinical hint suggesting autoimmune blistering dermatoses, be it pemphigus vulgaris, pemphigoid gestationis, or BP (9, 10). Then, the choice of appropriate laboratory diagnostics should not be difficult. Our case was diagnosed with a combination of imaging and biochemical-molecular techniques. The DIF visualised with the blue light-emitting diode technology-operated microscopy for patterning of IgG4 and IgG1 deposits along the DEJ in perilesional tissue combined with the multiplex ELISA detecting serum IgG antibodies to BP180 and/or BP230 is an approach advocated by us for routine differential laboratory diagnostics of BP in our native population (11, 12). Usually, we detect IgG4, but not IgG1 deposits with DIF in BP, nevertheless in our case IgG1, but not IgG4, deposits were detected (13). This suggests that the Th-1 immune response, conceivably induced by a viral trigger, predominated in our case over the Th-2 immune response characterizing BP in the elderly presenting itchy wheal-like lesions. The gradual maturation of the immune system during infancy (14) may, to some extent, underlie such a difference. Nevertheless, it is conceivable that inefficient regulation of the immune response in both senescence and infancy may be a shared pathological trait of BP in both the elderly and infants.

There are seven coronaviruses of varying pathogenicity that can infect humans (15). Autoimmune diseases concomitant with coronaviruses have been suggested to be associated with cross-reactivity of antibodies or activated lymphocytes with different antigens which own the same or similar epitopes (16). Two cases of pemphigus vulgaris following SARS-CoV-2 infection, as well as BP in an elderly female and a middle-aged female with COVID-19 were reported (17–20).

There are reports of BP in paediatric population being triggered by various vaccinations (21, 22). Moreover, linear IgA bullous dermatosis (LABD), an autoimmune blistering dermatosis sharing with BP the autoimmunity to the DEJ, can be induced by vaccinations in children (23). We diagnosed LABD developing right after simultaneous vaccinations with the Di-Te-Per, Act-HIB, and polio vaccines in an 18-month-old girl suggesting that there is a spectrum of living organisms-derived triggers of IgA-mediated autoimmune blistering dermatoses (24). Still, childhood vaccinations are unlikely to be a trigger for BP in our case as the boy was not vaccinated immediately before the occurrence of BP due to recurrent upper respiratory tract infections transmitted mainly by older brother of the boy from the nursery school. Therefore, COVID-19 remains the most likely trigger of BP, possibly in conjunction with the chain of other unspecified mucous membranes infections. It is plausible that BP developed in our infant following an immune response to the pathogen causing COVID-19 in relation to the familial exposure to this infectious agent. Nevertheless, a random association is also possible. Interestingly in this respect, a study on 414 individuals concluded that mRNA-based COVID-19 vaccinations can induce a spectrum of generally mild adverse skin rashes (25). Still, cases of BP following COVID-19 vaccinations were reported (26).

It is concluded that BP should be included in a differential diagnosis of infantile rashes showing blisters or vesicles or both as well as their prodromal and evolutionary lesions. Possible triggers, such as COVID-19, of BP in infancy should be identified and properly dealt with.

## Data Availability Statement

The raw data supporting the conclusions of this article will be made available by the authors, without undue reservation.

## Ethics Statement

Written informed consent was obtained from the minors' legal guardian/next of kin for the publication of any potentially identifiable images or data included in this article.

## Author Contributions

AR-W wrote the basis of the manuscript and delivered the patient. MJ and MB-D wrote sections of the manuscript and contributed to manuscript revision. MD contributed to conception, designed the article, and diagnosed the patient. All authors contributed to the article and approved the submitted version.

## Conflict of Interest

The authors declare that the research was conducted in the absence of any commercial or financial relationships that could be construed as a potential conflict of interest.

## Publisher's Note

All claims expressed in this article are solely those of the authors and do not necessarily represent those of their affiliated organizations, or those of the publisher, the editors and the reviewers. Any product that may be evaluated in this article, or claim that may be made by its manufacturer, is not guaranteed or endorsed by the publisher.
